# WDHD1 Leads to Cisplatin Resistance by Promoting MAPRE2 Ubiquitination in Lung Adenocarcinoma

**DOI:** 10.3389/fonc.2020.00461

**Published:** 2020-04-24

**Authors:** Lian Gong, Mengqing Xiao, Dong He, Yi Hu, Yuxing Zhu, Liang Xiang, Ying Bao, Xiaoming Liu, Qinghai Zeng, Jianye Liu, Ming Zhou, Yanhong Zhou, Yaxin Cheng, Yeyu Zhang, Liping Deng, Rongrong Zhu, Hua Lan, Ke Cao

**Affiliations:** ^1^Department of Oncology, Third Xiangya Hospital of Central South University, Changsha, China; ^2^Department of Respiratory, The Second People's Hospital of Hunan Province, Changsha, China; ^3^Department of Gastroenterology, Third Xiangya Hospital of Central South University, Changsha, China; ^4^Department of Dermatology, Third Xiangya Hospital of Central South University, Changsha, China; ^5^Department of Urology, Third Xiangya Hospital of Central South University, Changsha, China; ^6^Cancer Research Institute and Key Laboratory of Carcinogenesis of the Chinese Ministry of Health, Central South University, Changsha, China; ^7^Department of Gynaecology, Third Xiangya Hospital of Central South University, Changsha, China

**Keywords:** lung adenocarcinoma, cisplatin, drug sensitivity, ubiquitin ligase, WDHD1, MAPRE2

## Abstract

Ubiquitin ligases have been shown to regulate drug sensitivity. This study aimed to explore the role of the ubiquitin ligase WD repeat and HMG-box DNA binding protein 1 (WDHD1) in regulating cisplatin sensitivity in lung adenocarcinoma (LUAD). A quantitative analysis of the global proteome identified differential protein expression between LUAD A549 cells and the cisplatin-resistant strain A549/DDP. Public databases revealed the relationship between ubiquitin ligase expression and the prognosis of patients with LUAD. Quantitative real-time polymerase chain reaction and Western blotting were used to estimate the WDHD1 expression levels. Analysis of public databases predicted the substrate of WDHD1. Western blotting detected the effect of WDHD1 on microtubule-associated protein RP/EB family member 2 (MAPRE2) and DSTN. Functional analysis of MAPRE2 verified the interaction between WDHD1 and MAPRE2, as well as the interacting sites by methyl-thiazolyl-tetrazolium assay and flow cytometry, immunoprecipitation, protein stability, and immunofluorescence. Cell and animal experiments confirmed the effect of WDHD1 and MAPRE2 on cisplatin sensitivity in LUAD. Clinical data evaluated the impact of WDHD1 expression level on cisplatin sensitivity. Quantitative analysis of the global proteome revealed ubiquitin-dependent protein catabolism to be more active in A549/DDP cells than in A549 cells. WDHD1 expression was higher in A549/DDP cells than in A549 cells, and knocking out WDHD1 increased the sensitivity of A549/DDP cells to cisplatin. WDHD1 overexpression negatively correlated with the overall survival of LUAD patients. We observed that MAPRE2 was upregulated when WDHD1 was knocked out. A MAPRE2 knockout in A549 cells resulted in increased cell viability while decreasing apoptosis when the A549 cells exposed to cisplatin. WDHD1 and MAPRE2 were found to interact in the nucleus, and WDHD1 promoted the ubiquitination of MAPRE2. Following cisplatin exposure, the WDHD1 and MAPRE2 knockout groups facilitated cell proliferation and migration, inhibited apoptosis in A549/DDP cells, decreased apoptosis, and increased tumor size and growth rate in animal experiments. Immunohistochemistry showed that Ki67 levels increased, and levels of apoptotic indicators significantly decreased in the WDHD1 and MAPRE2 knockout groups. Clinical data confirmed that WDHD1 overexpression negatively correlated with cisplatin sensitivity. Thus, the ubiquitin ligase WDHD1 induces cisplatin resistance in LUAD by promoting MAPRE2 ubiquitination.

## Introduction

Lung cancer is the most common type of malignant tumor. It ranks first in morbidity and mortality globally, as well as in China, and is the leading cause of cancer-related deaths (1, 2). The treatment of lung cancer is achieved by a combination of various therapies, including surgery, chemotherapy, radiotherapy, molecular targeted therapy, and immunotherapy. As the early symptoms of lung cancer are not obvious, lung cancer is mostly diagnosed in the advanced stage. Therefore, chemotherapy continues to be an important treatment strategy for lung cancer. The chemotherapy regimen for lung cancer is based on platinum combined with other chemotherapeutic drugs, the most commonly used platinum drug being cisplatin (DDP). However, the failure of lung cancer treatment has typically occurred due to cisplatin resistance. Lung adenocarcinoma (LUAD) accounts for the highest proportion of lung cancer, ranging from 40 to 55%. Therefore, it is critical to explore the molecular mechanisms of cisplatin resistance in LUAD and provide a new basis for cisplatin sensitization in patients with LUAD.

Cisplatin resistance in lung cancer is closely related to DNA damage repair, apoptotic inactivation, activation of epithelial–mesenchymal transition, and characteristics of cancer stem cells (3–8). The related proteins and important molecules in the above signaling pathways are typically regulated by ubiquitination of proteins (9). Ubiquitin is a highly conserved molecule composed of 76 amino acids, which is widely expressed in eukaryotic organisms. The enzymes ubiquitin-activating enzyme (E1), ubiquitin-conjugating enzyme (E2), and ubiquitin ligase (E3) together modify a specific target protein by linking ubiquitin to it. E1 activates ubiquitin and transfers it to E2, and E3 recruits ubiquitinated E2, identifies substrates, and assists in the transfer of ubiquitin from E2 to protein substrates (10). E3 plays an important role in recognizing substrates during ubiquitination. Ubiquitin ligases have been shown to be associated with tumor development and a malignant phenotype. Moreover, previous studies have confirmed that E3 mediates cisplatin resistance through the regulation of various substrates.

Overexpression of the ubiquitin ligase MDM2 in malignant pleural mesothelioma is negatively correlated with patient prognosis and leads to p53 degradation and decreased cisplatin sensitivity (11). In addition, the ubiquitin ligase RNF31 is overexpressed in breast cancer tissues and MCF-7 cell lines, and it can promote polyubiquitination and degradation of p53 by stabilizing the ubiquitin ligase MDM2, which results in a reduction in cisplatin-induced apoptosis (12). The ubiquitin ligase NEDD4-1 promotes cisplatin resistance in lung cancer cells by inhibiting PTEN expression and activating Akt and its downstream proteins (13). Hakai (E3) is overexpressed in non–small cell lung cancer cell lines. Hakai interference leads to decreased expression of phosphorylated Akt, which significantly inhibits the growth of non–small cell lung cancer cells and enhances cisplatin drug sensitivity (14). Therefore, ubiquitin ligases can regulate key molecules of the signaling pathway to influence cisplatin resistance.

To explore the causes of cisplatin resistance in patients with LUAD, we screened the protein chip of both A549 and A549/DDP cell lines, as well as identified and analyzed any significantly altered proteins. The protein chip results demonstrated that ubiquitin-dependent protein catabolism was more active in A549/DDP cells than that in A549 cells. On the basis of existing literature and protein chip results, this study aims to investigate the role of ubiquitin ligase in regulating cisplatin sensitivity and its regulatory mechanism in LUAD. Our research may provide a scientific basis and novel insight for reversal of cisplatin resistance and personalized treatment of LUAD in future.

## Materials and Methods

### Cell Culture and Transfection

A549 and A549/DDP cell lines (Central South University, Changsha, China) were cultured in RPMI-1640 cell culture medium (Sigma-Aldrich, St. Louis, MO, USA) supplemented with 10% fetal calf serum, 100 U/mL penicillin, and 100 μg/mL streptomycin. The culture medium of the A549/DDP cells contained 2 μg/mL cisplatin (Solarbio Company, Beijing, China), in addition to the other components, and was incubated in 5% CO_2_ at 37°C. The purity of cisplatin was 98.5%. All RNA inhibitors and negative control siRNA were purchased from GenePharma (Shanghai, China). The siRNA targeting WD repeat and HMG-box DNA binding protein 1 (WDHD1) (15) (5′-GAUCAGACAUGUGCUAUUA-3′), ARPC1A (16) (5′-GUGGAGCACGACUCAUUUCTT-3′), and microtubule-associated protein RP/EB family member 2 (MAPRE2) (5′-UUGUUC–AGGAGCGGCCUAUTT-3′) were transfected into A549 and A549/DDP cells using Lipofectamine™ 2000 (Invitrogen Life Technologies, Carlsbad, California, USA) according to the manufacturer's instructions. After 24 h, the supernatant was replaced with culture medium, and the cells were incubated at 37°C in 5% CO_2_ for another 24 h.

### Quantitative Real-Time Polymerase Chain Reaction

Total RNA was extracted from A549 and A549/DDP cells using TRIzol, and reverse transcription was performed using the RevertAid First Strand cDNA Synthesis Kit (Thermo Fisher Science, Waltham, MA, USA). Real-time polymerase chain reaction (PCR) was performed using SYBR Green Premix PCR Master Mix (Roche, Mannheim, Germany). The relative quantification of WDHD1 and APRC1A was carried out according to the ^ΔΔ^C_T_ method. The primer sequences for amplification of WDHD1 were as follows: forward: 5′-AGGTCGTCCTAGACAGCG-3′; reverse: 5′-GCATGGGTCCATCATAAA-3′. The primer sequences for ARPC1A were as follows: forward: 5′-CAGTCCCAATAATCACGAA-3′; reverse: 5′-GGAGCCCAGTCAATACCT-3′. β-Actin was used as a housekeeping control. The primer sequences used were as follows: forward: 5′-CATTAAGGAGAAGCTGTGCT-3′; reverse: 5′-GTTGAAGGTAGTTTCGTGG–A-3′ (17).

### Western Blotting

The total protein in the cells and tissues was extracted using radioimmunoprecipitation assay (RIPA) buffer (Auragene, Changsha, China). The proteins were quantified using a BCA protein assay kit (Thermo Scientific). The protein samples were separated on a 10% sodium dodecyl sulfate–polyacrylamide gel electrophoresis (SDS-PAGE) gel, followed by transfer of the proteins to polyvinylidene fluoride membranes and blocking of the membranes using 5% Tris buffered saline with Tween-20 buffer containing 5% skim milk. Anti-MAPRE2 (1:100, ab45767; Abcam, Cambridge Science Park, UK), anti-WDHD1 (1:100, ab72436; Abcam), anti-DSTN (1:500, ab186754; Abcam), and anti-ubiquitin antibodies (1:200, 10201-2-AP; Proteintech, Wuhan, China) were used as primary antibodies and incubated with the membrane overnight at 4°C. The membrane was then washed and incubated with a secondary horseradish peroxidase–labeled goat anti–rabbit immunoglobulin G (IgG) antibody (1:4,000; Abcam) for 1 h at room temperature. Immunoassay was performed by enhanced chemiluminescence detection system (ECL; Cell Signaling Technology, Danvers, MA, USA) combined with Western blot system (Auragene). Strip signal strength was checked using the software IPP6.0. GAPDH (1:4,000, ab125247; Abcam) was used as an internal control to normalize the expression of other proteins.

### Bioinformatics Analysis

The public database DAVID (https://david.ncifcrf.gov/) was used to analyze the functional enrichment of significantly upregulated proteins. Searching for ubiquitin ligase and proteins interacting with ubiquitin ligase was performed through a website (http://ubibrowser.ncpsb.org/ubibrowser/home/index and http://iuucd.biocuckoo.org/). Public database Gepia, http://gepia.cancer-pku.cn/, was used to find the correlation between gene and prognosis. Searching gene expression was performed on a public database Ualcan (http://ualcan.path.uab.edu/index.html). By referring to the ubiquitination site prediction database Phosphosite, http://www.phosphosite.org, we can find proteins with ubiquitination sites.

### Methyl-Thiazolyl-Tetrazolium Assay

Cell viability was assessed using a methyl-thiazolyl-tetrazolium (MTT) assay (18). The A549 and A549/DDP cells were seeded in 96-well plates at a density of 1 × 10^4^ cells per well. The cells were incubated for 24 h following treatment with cisplatin [25% inhibitory concentration (IC25) concentration of cisplatin for A549/DDP cells = 20.240 μg/mL]. Then, 50 μL of 1 × MTT (Sigma-Aldrich) was added to each well according to the manufacturer's instructions and incubated at 37°C. After 4 h, MTT solution was removed, and 150 μL dimethyl sulfoxide was added to each well to dissolve the purple formazan crystals. The optical density of each well was measured at a wavelength of 570 nm using a microplate reader (Bio-Rad California, USA). Cell survival rate = OD of the test well/OD of the control well. GraphPad Prism 7.0 was used to create the resulting graph.

### Flow Cytometry Assay

Cellular apoptosis was evaluated by flow cytometry. The cells were treated with DDP. To assess apoptosis, an annexin V–[fluorescein isothiocyanate (FITC)]/PI apoptosis detection kit (KeyGEN Biotech, Nanjing, China) was used. After dual staining with FITC–annexin V and propidium iodide the level of fluorescence was measured by flow cytometry (FACS Canto II; BD, New Jersey, USA). Apoptosis rate = early apoptosis rate + late apoptosis rate.

### Coimmunoprecipitation

Si-con or si-WDHD1 was transfected into A549/DDP cells. After 24 h, the cells were lysed with RIPA (Auragene). Anti-MAPRE2 (1:100. ab45767; Abcam) and human IgG (1:150, bs-0297P; Bioss, Beijing, China) antibodies were added as precipitation antibodies and incubated overnight at 4°C. Then, 20 μL of protein A + G agarose was added and incubated for 2 h at 4°C. The protein-bead precipitations were denatured in SDS-PAGE by boiling for 5 min at 100°C, and a Western blot was performed to analyze the expression of ubiquitin, WDHD1, and MAPRE2 (19).

### Immunofluorescence Staining

The A549/DDP cells were grown until they reached 70% confluency. The cells were fixed with 4% paraformaldehyde stationary solution and incubated for 20 min, washed with phosphate-buffered saline (PBS) three times, and then treated with 1% Triton-x-100 for 10 min. The cells were pretreated with 10% normal goat serum for 30 min. The cells were incubated with the primary antibodies anti-WDHD1 (1:100, ab72436; Abcam) and anti-MAPRE2 (1:100, ab45767; Abcam) antibodies at 4°C overnight and then washed with PBS. The cells were then incubated with an Alexa Fluor® 555 goat anti-rabbit antibody diluted 1:100 in a blocking solution. Nuclei were stained with 4,6-diamidino-2-phenylindole for 15 min, and the cells were examined with Leica TCS-SP5 confocal laser microscope (Heidelberg, Germany).

### Wound Healing Assay

Approximately 5 × 10^5^ cells per well were seeded in a 24-well culture plate and treated with cisplatin. After incubation for 24 h, scratches were made using a sterile 20 μL pipette tip, and the plates were incubated at 37°C in 5% CO_2_. After incubation for 0 and 48 h, for each group, three fields with a wound area were chosen randomly and photographed under a microscope. The gap distance was quantitatively evaluated using ImageJ software. Relative mobility = (distance between the edges of migrated scratches/distance between the edges of initial scratches) ×100%.

### Colony Formation Assay

The transfected cells were cultured up to the logarithmic growth phase and then trypsinized and seeded into six-well plates (10^3^ cells per well). Cisplatin was added to the wells in accordance with the experimental design. Cells were incubated for 2–3 weeks at 37°C, 5% CO_2_. Cells were washed with PBS twice before harvest. The cells were fixed in 4% paraformaldehyde for 15 min, stained with hematoxylin, and counted under a microscope. The number of cells contained in each cell clone was counted, and the cell colony formation rate was calculated and photographed. The clone was counted if the cell number of the clone was at least 50. Clonal formation rate = number of clones/number of cells inoculated.

### Animal Experiments

All animal experiments were approved by the Animal Laboratory of Central South University and carried out in accordance with international guidelines and programs. Four- to 6-week-old male BALB/C nude mice (purchased from Hunan SJA Laboratory Animal Co., Ltd, Changsha, China) were subcutaneously injected with 1 × 10^6^ cells near the extremities of four limbs. When the mice developed palpable tumors, the mice were intraperitoneally administered with cisplatin (5 mg/kg; Solarbio Company) every week for 2 weeks. The size of the tumors was measured once every 3 days, six times in a row. All animals were sacrificed 25 days after inoculation, and the tumors were collected. The tumor tissues were photographed, and immunohistochemistry was performed. Tumor volume formula: *V* = *a* ^*^ *b*^2^^*^ 0.52 (mm^3^), where *a* is the longest diameter, and *b* is the shortest diameter of the tumor.

### TUNEL Staining

TUNEL staining was used to detect apoptotic tumor cells (20). The collected tumors were fixed in 4% paraformaldehyde solution for 60 min, embedded in paraffin, and cut into 3-μm sections. After being dewaxed and rehydrated, the sections were scrubbed with Tris-buffered saline buffer. Then, the sections were incubated with a mixture of TdT and dUTP at 37°C for 120 min following by the slides were treated with 0.3% H_2_O_2_ in methanol for 15 min. After being washed by PBS, the slides were added by converter-POD at 37°C for 30 min. Following incubation, excess labeling solution is washed off with PBS. 3,3′-Diaminobenzidine (DAB) was used to visualize cell apoptosis, and the DAB color was visualized under the microscope for ~15 min. Sections were then counterstained with hematoxylin, sealed with neutral gum, and finally examined under a microscope.

### Clinical Tissues

A total of 21 patients with LUAD receiving chemotherapy in the Third Xiangya Hospital (Changsha, China) from 2016 to 2018 were included in this study. The inclusion criteria were as follows: (1) histopathological examination confirming LUAD; (2) no indication of using molecular targeted drugs; and (3) no operation, or recurrence after operation, with assessable lesions. The 21 patients included in the study received cisplatin-combined chemotherapy and their sensitivity or resistance to cisplatin was determined by computed tomography (CT) analysis before and after cisplatin treatment. The 21 patients were divided into two groups: the cisplatin-sensitive group (*n* = 10) and the cisplatin-resistant group (*n* = 11). The responses to chemotherapy were scored using a tumor regression grade (TRG) developed by the American Joint Commission on Cancer and the College of American Pathology. We allocated the patients with a TRG of 0 or 1 to the cisplatin-sensitive group and those with TRG 2 or 3 to the cisplatin-sensitive group. The study was approved by the Research Ethics Committee of the Xiangya Third Hospital, and signed informed consent was obtained before each subject participated in the study.

### Immunohistochemistry Staining

First, paraffin-embedded tissues were sectioned, dewaxed, hydrated, and antigen-repaired. Next, 50 μL peroxidase-blocking solution and 50 μL non-immune animal serum were added, and the sections were incubated at room temperature for 10 min. The primary antibodies anti-WDHD1 (1:100, ab72436; Abcam) and anti-Ki67 (1:100, GTX16667; Genetex) were added to the sections and incubated overnight at 4°C. Each section, after washing, was incubated at room temperature for 30 min with a drop of biotin-labeled secondary antibody. 3,3′-Diaminobenzidine was used to develop the visual signal. Hematoxylin was used as a counterstain. Two pathologists who were blinded to clinical pathology information scored the samples. The score was determined by the proportion of positive tumor cells and the intensity of staining. Tumor cell proportions were scored as follows: “0” (< 5% positive tumor cells), “1” (5–25% positive tumor cells), “2” (25–50% positive tumor cells), “3” (25–75% positive tumor cells), and “4” (>75% positive tumor cells). Staining intensity was graded according to the following standard: “0” (no staining), “1” (weak staining = light yellow), “2” (moderate staining = yellow brown), and “3” (strong staining = brown). The total immunostaining score (scored as 0, 1, 2, 3, 4, 6, 8, 9, or 12) was calculated as the value of the proportion of positive cells score multiplied by the staining intensity score. Staining index scores ≥6 were identified as high expression, whereas scores <6 were considered low expression.

### Statistical Analysis

The data were statistically analyzed using GraphPad Prism 7.0 (GraphPad Inc., California, CA, USA) (21). All experiments were performed in triplicates, and data are expressed as mean ± standard deviation (Supplementary Material 2). The significance of the data sets was tested using analysis of variance (ANOVA). Comparison between specific groups was performed using a Student *t-*test (e.g., quantitative real-time PCR data). Multiple comparisons were performed using Bonferroni test and Tukey test (e.g., flow cytometry, wound healing assay, colony formation assay, and MTT assay). *p* < 0.05 in all cases was considered to be statistically significant.

## Results

### Analysis and Verification of the Global Proteome in A549 and A549/DDP Cells

#### Ubiquitin-Dependent Protein Catabolism Was More Active in A549/DDP Cells Than That in A549 Cells

**Figure 1:**
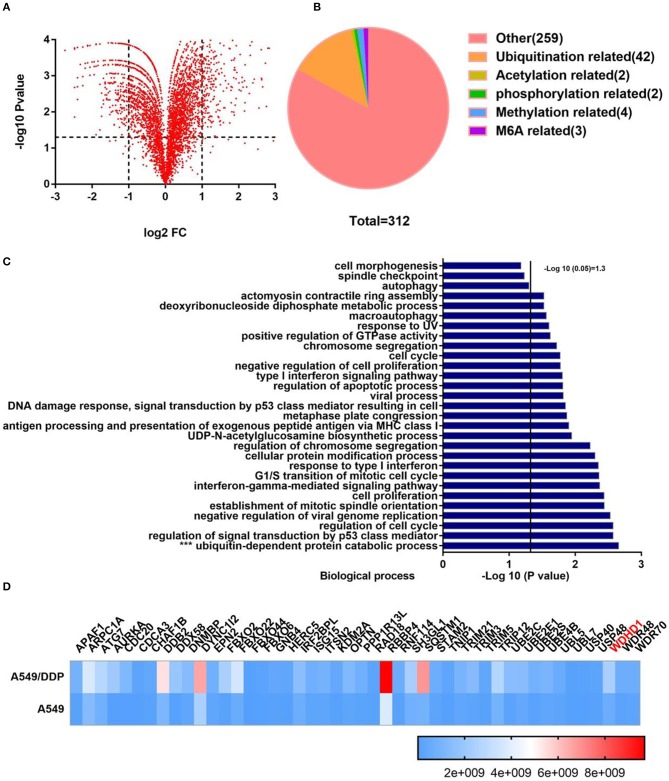
Quantitative analysis of the screened global proteome showed significant change in the protein expression in A549/DDP cells. **(A)** Quantitative analysis of the global proteome identified 5,758 quantitative proteins. Among the quantified proteins in A549/DDP cells, 312 proteins were upregulated, and 345 proteins were downregulated as compared to A549 cells. **(B)** Posttranslational modification-related proteins were significantly upregulated. **(C)** According to an enrichment analysis, ubiquitin-dependent protein catabolism was active. **(D)** The protein abundance and relative expression of 46 ubiquitin-related enzymes in A549/DDP and A549 cells.

Cisplatin resistance in patients with LUAD is one of the main reasons for poor tumor prognosis. To explore the mechanism of cisplatin resistance in LUAD, we performed a quantitative analysis of the global proteome of both A549/DDP and A549 cell lines. A total of 7,475 protein groups were identified, among which 5,758 proteins were quantified. The fold-change cutoff was set when proteins with quantitative ratios >2 or <1/2 were deemed significant (*p* < 0.05). Among the quantified proteins in A549/DDP cells, we found that 312 proteins were upregulated, and 345 proteins were downregulated as compared to A549 cells (Figure 1A). Analysis of the significantly upregulated proteins revealed that post-translational modification of related proteins were upregulated (Figure 1B). The public database, https://david.ncifcrf.gov/, was used to analyze the functional enrichment of significantly upregulated proteins. We found that ubiquitin-dependent protein catabolism was more active in A549/DDP cells as compared to that in A549 cells, with statistically significant differences (*p* < 0.05) (Figure 1C). Ubiquitination-related websites (http://ubibrowser.ncpsb.org/ubibrowser/home/index and http://iuucd.biocuckoo.org/) and protein chip results suggested that 46 ubiquitination-related enzymes were significantly upregulated in A549/DDP cells, with protein abundance as shown in the heat map (Figure 1D). These results suggest that protein ubiquitination is associated with cisplatin resistance.

#### Knockout of the Ubiquitin Ligase WDHD1 Increases Cisplatin Sensitivity in LUAD Cells

**Figure 2:**
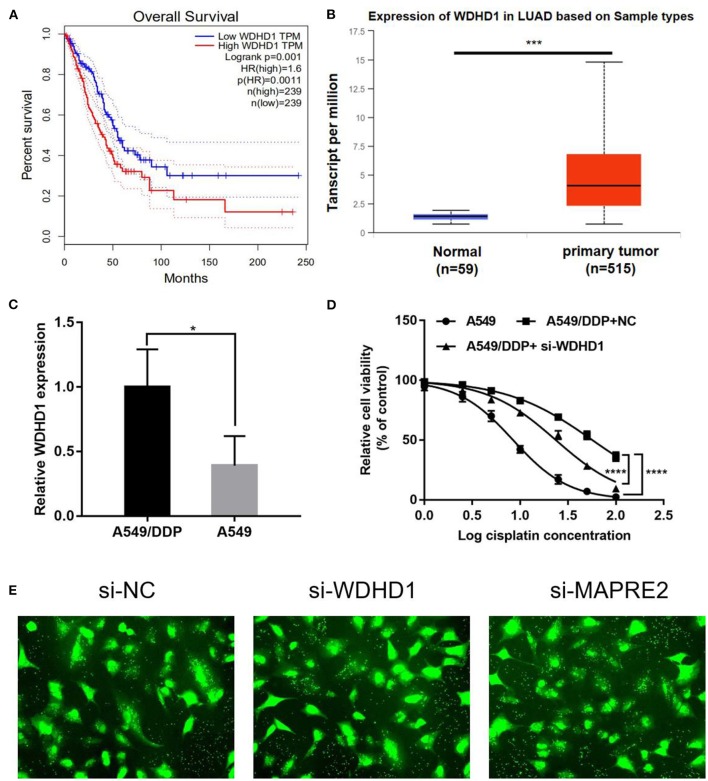
WDHD1 knockout increases cisplatin sensitivity in lung adenocarcinoma cells. **(A)** According to the public database, overexpression of WDHD1 negatively correlated with the prognosis of patients with lung adenocarcinoma. **(B)** Referring to the public database, WDHD1 expression in lung cancer tissues was higher than that in adjacent tissues. **(C)** The PCR results showed that WDHD1 expression in A549/DDP cells was significantly higher than that in A549 cells. **(D)** After WDHD1 was knocked out, the proliferation and IC50 of A549/DDP decreased significantly. **(E)** The transfection efficiency of siRNA. *n* = 3; ^*^p < 0.05; ^***^p < 0.001; ^****^p < 0.0001.

Ubiquitin ligases play an important role in recognizing substrates during protein ubiquitination and are closely associated with cisplatin resistance in malignant tumors. Therefore, to understand the role of ubiquitin ligases in cisplatin resistance in LUAD, we consulted the public database http://gepia.cancer-pku.cn/ and found that the upregulation of the ubiquitin ligase WDHD1 (Figure 2A) and ARPC1A (Supplementary Figure 1A) negatively correlated with patient prognosis. The expression of WDHD1 in the A549/DDP cell line was 2.17 times higher than that in the A549 cell line in a quantitative analysis of the global proteome. Therefore, we speculated that WDHD1 overexpression might be the cause of cisplatin resistance in LUAD. According to the public database http://ualcan.path.uab.edu/index.html, the expression of WDHD1 (Figure 2B) and ARPC1A (Supplementary Figure 1B) in lung cancer tissues was higher than that in the adjacent tissues, suggesting that the upregulation of WDHD1 and ARPC1A may be one of the reasons for the occurrence and development of LUAD. The level of WDHD1 and ARPC1A expression in A549/DDP and A549 cells was detected by PCR (Supplementary Figure 1C). The results showed that the level of WDHD1 expression in A549/DDP cell lines was higher than that in the A549 cell lines (*P* < 0.05), which was consistent with the public database and the trend of the protein chip results (Figure 2C). To further explore the effect of WDHD1 on the sensitivity of A549/DDP cells to cisplatin, we constructed siRNA to knock out WDHD1 in A549/DDP cells. MTT assay was used to detect the viability of tumor cells and calculate the IC50 and IC25 for cisplatin toward A549/DDP cells. It was found that the viability of A549/DDP cells significantly decreased after WDHD1 knockout (Figure 2D), and the IC50 decreased significantly (Supplementary Table 1).

### WDHD1 Increases Cisplatin Resistance by Promoting MAPRE2 Ubiquitination in LUAD

#### WDHD1 Decreases the Expression of MAPRE2 in A549/DDP Cells

Literature suggests that ubiquitin ligases affect the sensitivity of malignant tumor cells to cisplatin primarily via regulating the expression of various substrates. To further explore the specific mechanism of WDHD1-induced cisplatin resistance in LUAD cells, we aimed to identify the downstream target proteins of WDHD1. Because the main function of ubiquitination is to degrade the target proteins after ubiquitination, the downstream target protein should be downregulated and interact with WDHD1. Combining a protein chip and the protein interaction database http://iuucd.biocuckoo.org, 15 proteins were found to be downregulated by 1.5 times or more and interact with WDHD1. Moreover, the ubiquitin ligase target proteins must have ubiquitination sites to be ubiquitinated. By referring to the ubiquitination site prediction database http://www.phosphosite.org, it was suggested that there are seven proteins with ubiquitination sites in the aforementioned downregulated proteins (Supplementary Table 2). Combined with the http://ualcan.path.uab.edu/index.html public database, it is suggested that only MAPRE2 and DSTN are expressed at low levels in LUAD (Supplementary Figure 2A). Therefore, we speculate that MAPRE2 and DSTN may be the target proteins of WDHD1.

**Figure 3:**
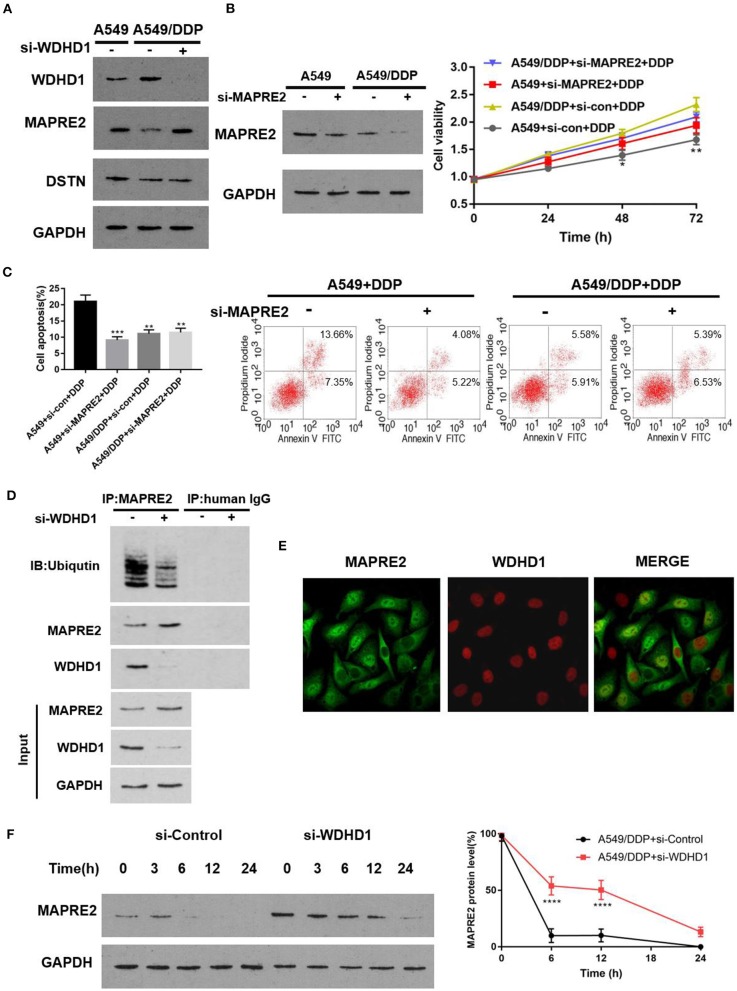
WDHD1 forms a complex with MAPRE2 and increases the ubiquitination degradation of MAPRE2. **(A)** Western blot results indicating that WDHD1 expression was higher in A549/DDP cells than in A549 cells. MAPRE2 was upregulated, and DSTN did not significantly change after knocking out WDHD1. **(B,C)** The results from an MTT assay and flow cytometry showing that after MAPRE2 was knocked out in A549 cells, the drug sensitivity of lung adenocarcinoma cells to cisplatin decreased, viability increased, and apoptosis decreased. **(D)** Coimmunoprecipitation showing that WDHD1 and MAPRE2 are interacting proteins. **(E)** Immunofluorescence demonstrating that WDHD1 (red) is primarily present in the nucleus, and MAPRE2 (green) is present in both nucleus and cytoplasm. **(F)** Protein stability experiments confirming that MAPRE2 degradation slowed down after knocking out WDHD1. DDP: IC25 concentration of cisplatin for A549/DDP cells. *n* = 3; ^*^p < 0.05; ^**^p < 0.01; ^***^p < 0.001; ^****^p < 0.0001.

To determine whether WDHD1 can regulate the expression of MAPRE2 and DSTN, the expression of MAPRE2 and DSTN in A549/DDP cells was detected by Western blotting (WB) after knocking out WDHD1 with si-WDHD1. The transfection efficiency of siRNA is shown in Figure 2E. The results showed that the knocking out of WDHD1 could increase the expression of MAPRE2 but had no significant effect on DSTN (Figure 3A and Supplementary Figure 2B). The WB results showed that WDHD1 expression in A549/DDP cells was higher than that in A549 cells (Figure 3A). These results were consistent with the protein chip findings, which confirmed the reliability of the chip. To explore whether the downregulation of MAPRE2 can affect the cisplatin sensitivity of LUAD cells, we constructed a siRNA knockout of MAPRE2 (si-MAPRE2) and control si-RNA (si-con). Both A549 and A549/DDP cells were treated with cisplatin at an IC25 concentration of cisplatin for A549/DDP cells and transfected with Si-Con and si-MAPRE2, respectively. A WB confirmed that MAPRE2 was successfully knocked out, and after MAPRE2 knocked out, A549 cell viability increased (Figure 3B). Flow cytometry results indicated that apoptosis was decreased under the action of cisplatin after MAPRE2 was knocked out in A549 and A549/DDP cells (Figure 3C). These results suggest that a decrease in MAPRE2 may be the cause of cisplatin resistance in LUAD cells. In conclusion, we speculate that MAPRE2 may act as a downstream target protein of WDHD1 and plays an important role in regulating cellular sensitivity to cisplatin.

#### WDHD1 Induces Cisplatin Resistance in A549/DDP Cells by Increasing the Ubiquitination Degradation of MAPRE2

Protein molecules perform essential cellular functions by forming protein complexes. To determine whether WDHD1 regulates the expression of MAPRE2 by forming protein complexes, we investigated the interaction between the ubiquitin ligase WDHD1 and MAPRE2 by coimmunoprecipitation (Co-IP) and detected the level of MAPRE2 ubiquitination. A549/DDP cells transfected with either si-con or si-WDHD1 were established as groups A and B, respectively. Coimmunoprecipitation was performed after successful transfection. The results showed that WDHD1 and MAPRE2 interacted with each other, and the level of MAPRE2 ubiquitination was significantly decreased when WDHD1 was knocked out (Figure 3D). The immunofluorescence results showed that WDHD1 (red) primarily existed in the nucleus, and MAPRE2 (green) existed both in the nucleus and cytoplasm, suggesting that the interaction between WDHD1 and MAPRE2 mainly occurred in the nucleus (Figure 3E). The protein stability test confirmed the effect of WDHD1 on MAPRE2 degradation. The results showed that the rate of MAPRE2 degradation was significantly reduced when WDHD1 was knocked out (Figure 3F). The above experiments confirmed that WDHD1 and MAPRE2 interact with each other, and WDHD1 promotes the degradation of MAPRE2 by ubiquitination.

**Figure 4:**
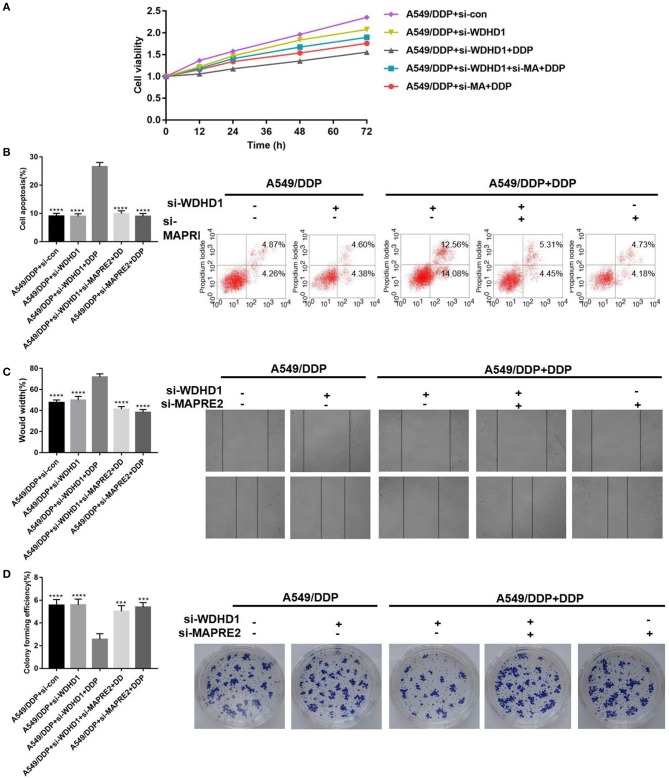
WDHD1 leads to cisplatin resistance by increasing MAPRE2 ubiquitination. **(A)** MTT assay, **(B)** flow cytometry, **(C)** scratch assay, and **(D)** cell colony formation assay results confirming that WDHD1 induced cisplatin resistance in lung adenocarcinoma by downregulating MAPRE2. *n* = 3; ^***^p < 0.001; ^****^p < 0.0001.

A functional recovery experiment was conducted to explore the effect of WDHD1 on cisplatin resistance by regulating MAPRE2, by establishing five groups designated A to E. Groups A was transfected with si-control; groups B and C were transfected with si-WDHD1; group D was transfected with both si-WDHD1 and si-MAPRE2; and group E was transfected with si-MAPRE2. Groups C to E were treated with DDP. After the DPP treatment, MTT assay (Figure 4A), flow cytometry (Figure 4B), scratch test (Figure 4C), and cell clone formation experiment (Figure 4D) were performed. The results showed that the cellular viability decreased, and apoptosis increased significantly after knocking out WDHD1 in A549/DDP cells. When both WDHD1 and MAPRE2 were knocked out, cellular viability increased, and apoptosis decreased.

**Figure 5:**
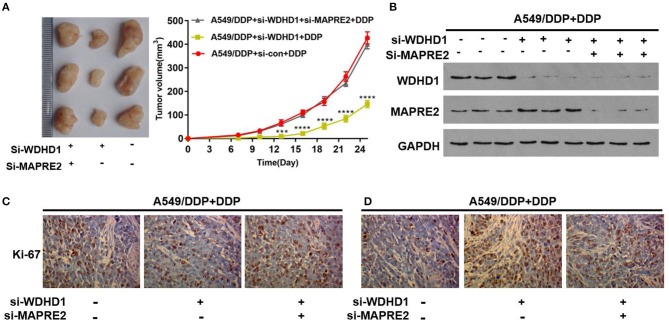
Verification of the function of WDHD1 by animal experiments. **(A)** The transplanted tumor experiment showing that the inhibition of tumor volume by cisplatin in WDHD1 knockout group was more obvious than that in the control group. **(B)** A Western blot indicating that WDHD1 expression was upregulated in mice tissues implanted with A549/DDP cells, and MAPRE2 expression was increased in tumor tissues implanted with WDHD1 knockout cells. **(C,D)** The expression of Ki67 in the A549/DDP WDHD1 knockout group was significantly lower than that in the control group. The apoptotic index detected by a TUNEL assay was significantly higher in A549/DDP WDHD1 knockout group than that in the control group. ^***^p < 0.001; ^****^p < 0.0001. DDP: IC25 concentration for A549/DDP cells.

**Figure 6:**
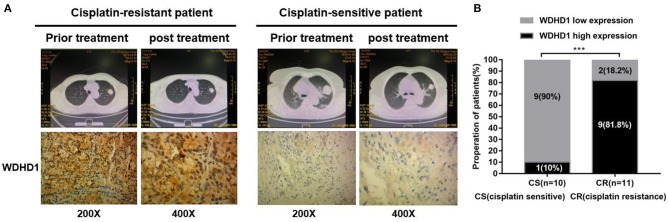
The expression of WDHD1 in cisplatin-resistant lung adenocarcinoma tissues is higher than that in cisplatin-sensitive resistant tissues. **(A)** WDHD1 expression in paraffin-embedded cisplatin-treated lung adenocarcinoma tissues as measured by *in situ* hybridization. Representative cases of cisplatin-sensitive (*n* = 10) and cisplatin-resistant (*n* = 11) patients with low or high WDHD1 expression are shown. **(B)** Proportion of cisplatin-treated lung adenocarcinoma patients with low or high WDHD1 expression. ^***^p < 0.001.

Animal experiments were conducted to verify the effect of WDHD1 and MAPRE2 interaction on cisplatin drug sensitivity. In groups A and B, A549/DDP cells were transfected with si-control and si-WDHD1, respectively. Group C was transfected with both si-WDHD1 and si-MAPRE2. After successful transfection, cells in groups A to C were subcutaneously implanted into mice. When the mice developed palpable tumors, they were administered cisplatin. We found that in comparison with group B the growth rate of the tumors in group C was significantly higher (Figure 5A). A WB confirmed that WDHD1 expression was upregulated in A549/DDP tumor tissues, and MAPRE2 was upregulated in WDHD1 knockout tumor tissues (Figure 5B). The expression of Ki67 in the WDHD1 knockout group was significantly lower than that in the control group (Figure 5C). The TUNEL assay results showed that the apoptotic index of the WDHD1 knockout group was significantly increased (Figure 5D). Combined with clinical CT and pathological analysis (Figures 6A,B), it could be concluded that cisplatin resistance was increased in patients with WDHD1 overexpression. The *in vivo* experiments further demonstrated that WDHD1 knockout increased the sensitivity of LUAD to cisplatin, decreased the proliferation of LUAD cells, and increased apoptosis.

Therefore, cell, animal, and clinical experiments confirmed that upregulation of the ubiquitin ligase WDHD1 can increase the ubiquitination degradation of MAPRE2, leading to cisplatin resistance in LUAD. Thus, WDHD1 may be an important molecular target for the reversal of cisplatin resistance in LUAD patients.

## Discussion

In an attempt to explore the specific molecular mechanism of cisplatin resistance in LUAD, this study identified differentially expressed proteins in A549/DDP and A549 cells by a quantitative analysis of the global proteome. The significantly upregulated proteins was enriched by the public database David in A549/DDP compared to A549 cells, and we found that ubiquitin-dependent protein catabolism is active in A549/DDP cells. A total of 42 ubiquitin ligases were identified among the significantly upregulated proteins. Therefore, we hypothesized that abnormal protein ubiquitination may be the cause of cisplatin resistance in LUAD. To date, studies have shown that cisplatin resistance is related to the regulation of ubiquitin ligase. For example, the ubiquitin ligase TRIM65 is highly expressed in non–small cell lung cancer, and cellular experiments have confirmed that it can reduce cisplatin-induced apoptosis by promoting p53 polyubiquitination degradation (22). The ubiquitin ligase TRIM37 has been shown to ubiquitinate adenylate kinase NEMO and activate the nuclear factor κB signaling pathway, which makes esophageal cancer cells cisplatin-resistant. Literature suggests that ubiquitin ligase primarily regulates cisplatin drug sensitivity by recognizing and regulating its substrates (23). Therefore, E3 was the main target of our present study. The public databases suggested that expression of WDHD1 and ARPC1A is increased in lung cancer tissues as compared to adjacent tissues, which negatively correlated with patient prognosis, suggesting that the upregulation of WDHD1 and ARPC1A may be one of the reasons for the occurrence and development of LUAD. Polymerase chain reaction and WB results demonstrated higher expression of WDHD1 in A549/DDP cells than in A549 cells. Moreover, the results of MTT assay revealed that A549/DDP cell viability, and IC50 decreased significantly when WDHD1 was knocked out. Therefore, we speculate that WDHD1 leads to cisplatin resistance in LUAD cells and present a preliminary exploration of the mechanism of this drug resistance.

WDHD1, also known as CTF4 or AND-1, is a DNA-binding protein in the nucleoplasm (24) that binds to DNA through the HMG domain (25). Moreover, WDHD1 directly interferes with the formation and progression of replication forks or indirectly interferes with DNA replication by stabilizing DNA helicase complex CMG (26) and interacting with DNA polymerase I (polA) (27), which is required for initiation of DNA replication (28, 29). In addition, studies have shown that WDHD1 regulates the cellular response to DNA damage (30, 31), DNA repair, and mitosis (32). WDHD1 has also been reported to be closely related to malignant tumors. For example, studies have confirmed that WDHD1 is upregulated in cervical cancer cells, and knocking out WDHD1 in these cells leads to G1 stagnation, which affects the cell cycle and cellular replication (33). Similarly, high WDHD1 expression was found to be negatively correlated with non–small cell lung cancer and esophageal squamous cell carcinoma, and the knockout of WDHD1 could effectively inhibit the growth of both lung and esophageal cancer cells (15); however, no studies have reported the association between WDHD1 and cisplatin sensitivity in LUAD.

On the basis of the quantitative analysis of the global proteome and the public websites, Biocuckoo, Phosphosite, and Ualcan, we speculate that MAPRE2 and DSTN may be the target proteins of WDHD1. In this study, the WB results showed that MAPRE2 was upregulated, and DSTN did not change significantly when WDHD1 was knocked out. Moreover, the MTT assay revealed that the MAPRE2 knockout was associated with increased cell viability and decreased apoptosis in A549 cells, suggesting that decreased MAPRE2 may be the cause of cisplatin resistance in LUAD. MAPRE2 is a microtubule-associated protein involved in microtubule polymerization, which is also essential for spindle symmetry during mitosis (34). Literature reveals that MAPRE2 is associated with the occurrence and development of malignant tumors. It has been reported that MAPRE2 is overexpressed in hepatocellular carcinoma (35) and esophageal squamous cell carcinoma (36); thus, MAPRE2 might be involved in tumorigenesis and promotion of tumor cell growth through Wnt signaling pathway or Aurora-B activation (37, 38). A study by Abiatari et al. (39) demonstrated that overexpression of MAPRE2 is associated with decreased survival and perineural infiltration in pancreatic cancer patients. Therefore, MAPRE2 overexpression in various malignant tumors is positively correlated with tumor growth, nerve infiltration, and poor prognosis. However, using the public database ULACN, it was observed that the levels of MAPRE2 expression in different types of malignant tumors and normal tissues differ from those suggested in the literature. For example, in LUAD, renal papillary carcinoma, bladder urothelial carcinoma, and other cancers, MAPRE2 expression is significantly lower than that in normal tissues. Therefore, MAPRE2 expression may be tissue-specific and plays a differential role in various malignant tumors. According to the protein chip and experimental results, it has been demonstrated that low MAPRE2 expression in LUAD cells may be a contributing factor to cisplatin resistance in LUAD. Moreover, Co-IP results showed that there was interaction between WDHD1 and MAPRE2. Further, immunofluorescence results revealed that WDHD1 interacts with MAPRE2 in the nucleus. Protein stability test results indicated that WDHD1 promotes ubiquitination degradation of MAPRE2. In addition, both functional recovery and animal experiments confirmed that WDHD1 could induce cisplatin resistance by increasing the ubiquitination degradation of MAPRE2. Finally, clinical CT and pathological analysis further confirmed that WDHD1 overexpression increased cisplatin resistance in patients with LUAD.

In conclusion, our results indicate that the ubiquitin ligase WDHD1 induces cisplatin resistance in LUAD by promoting the degradation of MAPRE2. These findings suggest that WDHD1 and MAPRE2 may be potential biomarkers of cisplatin sensitivity in patients with LUAD. Although the regulatory mechanism of ubiquitin ligase in tumorigenesis is highly complex, our results indicate that the level of ubiquitin ligase expression and its substrates has the potential to predict cisplatin sensitivity in LUAD. Although cellular, animal, and clinical data were presented in this study, something remain poorly understood, for example, effect of overexpression of MAPRE2 in A549 on cisplatin sensitivity, specific mechanism of MAPRE2 regulating cisplatin drug sensitivity, and the specific sites and types of ubiquitination. To explore the specific mechanism of MAPRE2 regulating cisplatin drug sensitivity, we use the public database https://string-db.org/ to explore MAPRE2′ interaction protein (Supplementary Figure 3A) and search their expression level and change through our protein chip (Supplementary Figure 3B). Among them, AURKB is significantly high expression in A549/DDP, and it has been confirmed that its high expression is related to cisplatin resistance (40, 41). Then, DNA damage repair gene set was obtained from GSEA, and the correlation between MAPRE2 and the DNA damage repair gene was analyzed by String. Two proteins were related to MAPRE2 (Supplementary Figure 3C). RAE1 was highly expressed in A549/DDP, and its high expression was negatively related to the prognosis of LUAD patients (Supplementary Figure 3D). MAPRE2 may affect cisplatin drug sensitivity by regulating AURKB and DNA damage repair–related proteins. Thus, we will overexpress MAPRE2 in A549/DDP to further confirm its effect on cisplatin drug sensitivity and explore the specific mechanism of MAPRE2 affecting cisplatin drug sensitivity from the perspective of DNA damage repair and AURKB regulation in our further experiment. In addition, future studies should focus on understanding the specific sites and types of ubiquitination. Moreover, further exploration of the relationship between ubiquitin ligase and cisplatin sensitivity in LUAD will promote a better understanding of tumor biology. It is important to consider combining the basic factors of cancer biology with clinical practice, which will provide new ideas for the clinical reversal of drug resistance in LUAD, and promote the development of effective treatment strategies.

In summary, this study demonstrates that the ubiquitin ligase WDHD1 is overexpressed in LUAD and plays an important role in cisplatin resistance by promoting MAPRE2 ubiquitination. Our findings indicated a novel molecular mechanism underlying cisplatin resistance in LUAD. Thus, WDHD1 and MAPRE2 could serve as novel therapeutic targets for reversal of cisplatin resistance in LUAD.

## Data Availability Statement

The raw data supporting the conclusions of this article will be made available by the authors, without undue reservation, to any qualified researcher.

## Ethics Statement

The studies involving human participants were reviewed and approved by the Ethics Committee of the Third Xiangya Hospital of Central South University. The patients/participants provided their written informed consent to participate in this study. The animal study was reviewed and approved by Ethics Committee for the Animal Laboratory of Central South University. Written informed consent was obtained from the individual(s) for the publication of any potentially identifiable images or data included in this article.

## Author Contributions

LG and KC conceived the study and performed data interpretation. LG and MX performed the experiments, analyzed data, and wrote the manuscript. DH, YH, YZhu, LX, YB, XL, QZ, JL, MZ, YZho, YC, YZha, LD, RZ, and HL participated in the experiments.

## Conflict of Interest

The authors declare that the research was conducted in the absence of any commercial or financial relationships that could be construed as a potential conflict of interest.

## Abbreviations

E1: ubiquitin-activating enzyme
E2: ubiquitin-conjugating enzyme
E3: ubiquitin ligase
WDHD1: WD repeat and HMG-box DNA binding protein 1
MAPRE2: microtubule-associated protein RP/EB family member 2
LUAD: lung adenocarcinoma
CO-IP: CO-immunocoprecipitation
IHC: immunohistochemical
PCR: polymerase chain reaction
siRNA: small interfering RNA.
