# Supplementary figures and images for: WDHD1 Leads to Cisplatin Resistance by Promoting MAPRE2 Ubiquitination in Lung Adenocarcinoma
Source: Front Oncol. 2020 Apr 24;10:461. doi: 10.3389/fonc.2020.00461 (PMC7212426; doi:10.3389/fonc.2020.00461)

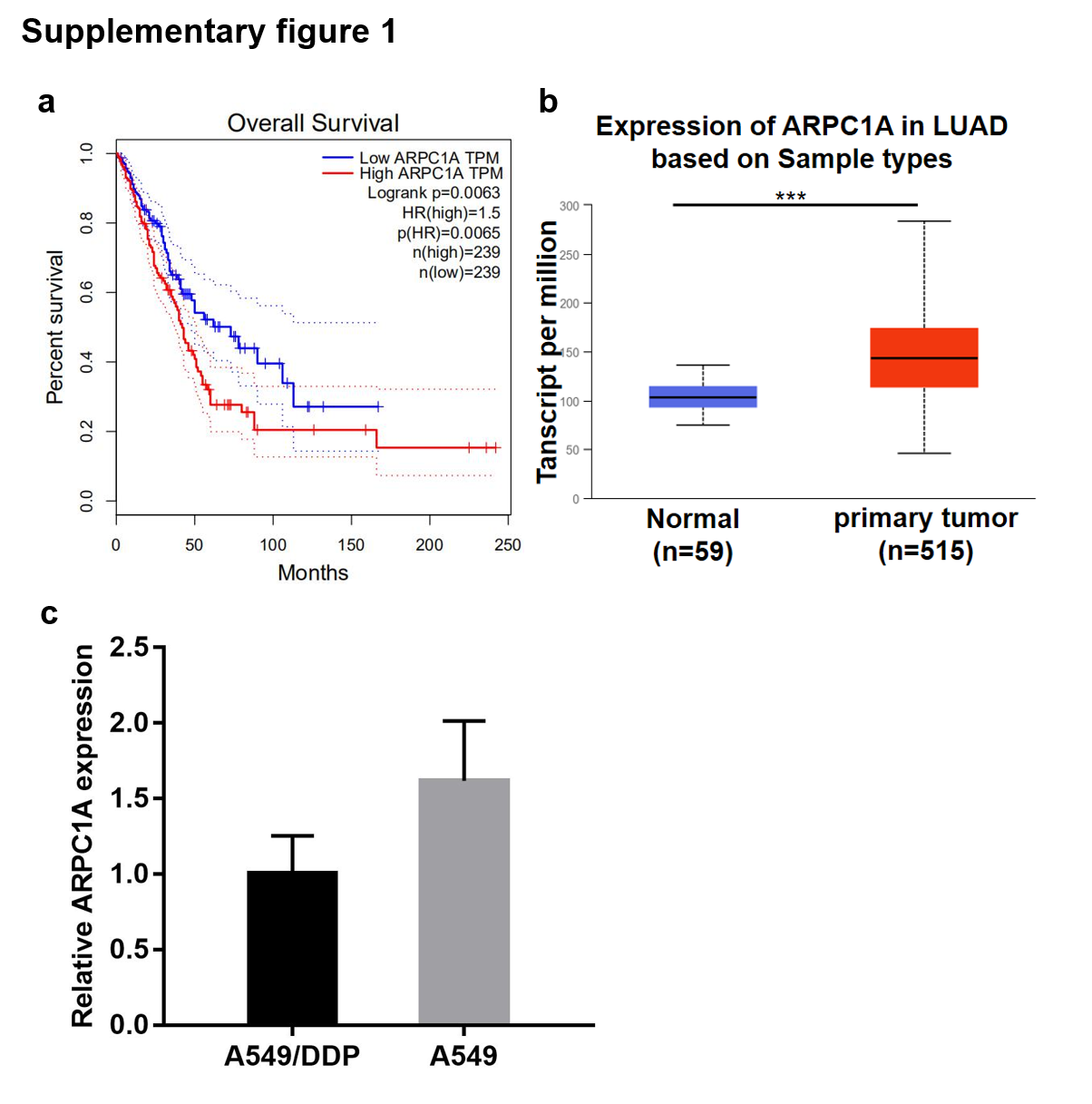

Supplement: Supplementary file 1 [file Image_1.TIF]

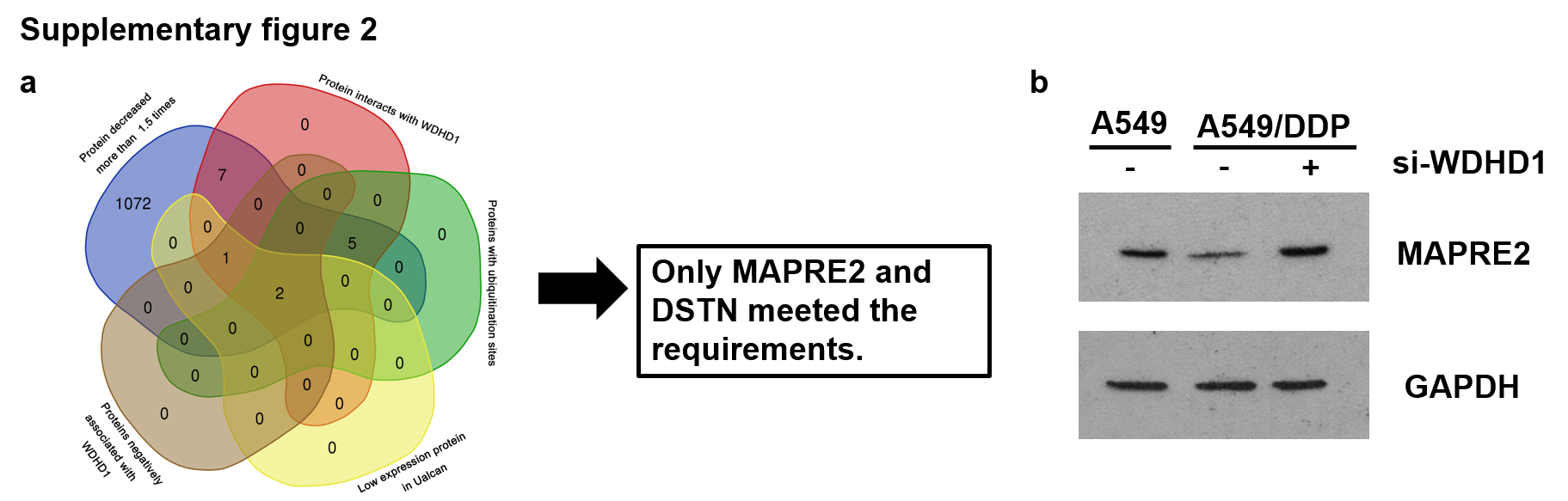

Supplement: Supplementary file 2 [file Image_2.TIF]

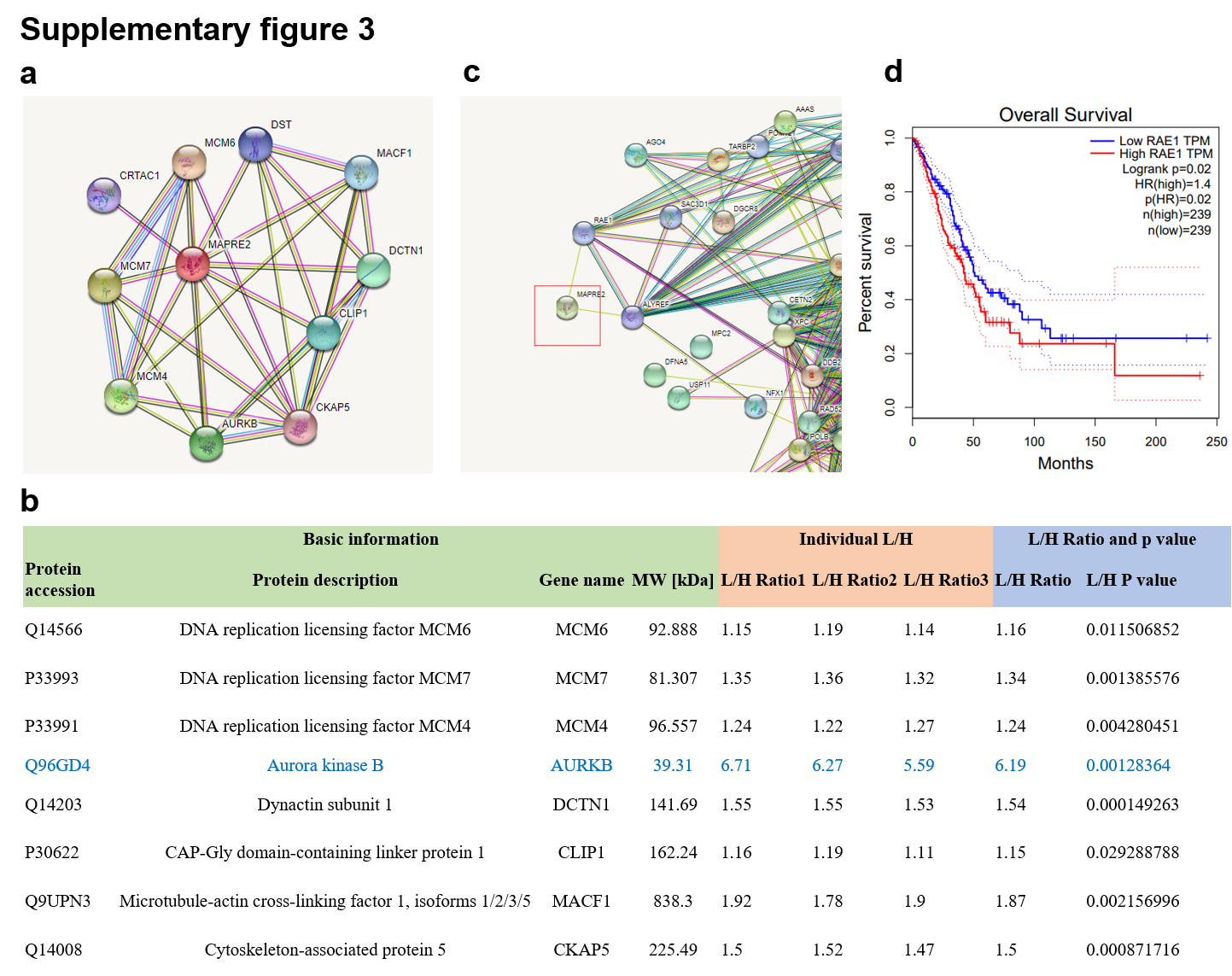

Supplement: Supplementary file 3 [file Image_3.TIF]
