# Supplementary material for: WDHD1 Leads to Cisplatin Resistance by Promoting MAPRE2 Ubiquitination in Lung Adenocarcinoma
Source: Front Oncol. 2020 Apr 24;10:461. doi: 10.3389/fonc.2020.00461 (PMC7212426; doi:10.3389/fonc.2020.00461)
Supplement: Supplementary file 4 [file Data_Sheet_1.pdf]

Supplementary Material 1

Supplementary figure 1

a) The expression of ARPC1A in lung cancer tissues was higher than that in the adjacent tissues. b) The high expression of ARPC1A was negatively correlated with the prognosis of patients with lung adenocarcinoma. c) The PCR results showed that ARPC1A expression in A549/DDP cells was lower than that in A549 cells.

Supplementary figure 2

a) Access to public data indicates that MAPRE2 and DSTN meet the substrate requirements of WDHD1. b) MAPRE2 was upregulated significantly after knocking out WDHD1.

Supplementary figure 3

a) Search MAPRE2's interacting protein. b) Changes of interacting proteins in protein chip. c) The relationship between DNA damage repair gene and MAPRE2 d) RAE1 expression and prognosis

Supplementary Table 1. 95% confidence interval of IC50

	A549+si-con	A549/DDP+si-con	A549/DDP+si-WDHD1
μg/mL	7.93 to 9.01	52.93 to 61.14	21.36 to 26.07

Supplementary Table 2. Prediction of MAPRE2 and DSTN ubiquitination sites

K119-ub	HEYIHNFkLLQAsFk
K126-ub	kLLQAsFkRMNVDkV
K132-ub	FkRMNVDkVIPVEkL
K138-ub	DkVIPVEkLVKGRFQ
K155-ub	LDFIQWFkkFYDANy
K156-ub	DFIQWFkkFYDANyD
K165-ub	YDANyDGkEyDPVEA
K193-ub	EQIFNLPkkSHHANs
K194-ub	QIFNLPkkSHHANsP
K207-ub	sPTAGAAkssPAAkP
K213-ub	AkssPAAkPGstPsR
K235-ub	SSSGSASksDkDLET
K238-ub	GSASksDkDLETQVI
K255-ub	NEQVHSLkLALEGVE
K263-ub	LALEGVEkERDFYFG

K271- ub	ERDFYFGkLREIELL

Prediction of DSTN ubiquitination sites

K19-ub	CRIFyDMkVRkCstP
K22-ub	FyDMkVRkCstPEEI
K30-ub	CstPEEIkKRKKAVI
K45-ub	FCLsADkkCIIVEEG
K78-ub	FVGMLPEkDCRYALy
K92-ub	yDAsFETkESRKEEL
K112-ub	APELAPLkSkMIyAS
K114-ub	ELAPLkSkMIyASSk
K121-ub	kMIyASSkDAIkKkF
K127-ub	SkDAIkKkFQGIkHE
K132-ub	kKkFQGIkHECQANG
