# Supplementary material for: WDHD1 Leads to Cisplatin Resistance by Promoting MAPRE2 Ubiquitination in Lung Adenocarcinoma
Source: Front Oncol. 2020 Apr 24;10:461. doi: 10.3389/fonc.2020.00461 (PMC7212426; doi:10.3389/fonc.2020.00461)
Supplement: Supplementary file 5 [file Data_Sheet_2.pdf]

Supplementary Material 2

WDHD1 PCR(figure 2c)

A549/DDP	A549
1±0.292	0.39±0.2291

Dose-response curve(figure 2d)

	A549	A549/DDP+NC	A549/DDP+ si-WDHD1
	100	100	100
1	94.73±3.349	98.65±0.374	98.36±2.892
2.5	86.23±4.219	96.21±1.135	91.23±1.351
5	70.05±4.365	91.19±2.712	83.67±2.31
10	42.21±3.038	82.95±2.289	72.76±2.416
25	17.04±3.764	69.13±2.172	54.2±3.507
50	7.059±1.737	53.95±3.385	28.3±1.987
100	2.426±1.13	36.03±3.73	9.454±1.731

MAPRE2 MTT(figure 3b)

	A549+si-con+DDP	A549+si-MAPRE2+DDP	A549/DDP+si-con+DDP	A549/DDP+si-MAPRE2+DDP
0	0.9483±0.0422	0.954±0.03819	0.9404±0.03024	0.9555±0.02602
24	1.15±0.05258	1.271±0.0837	1.416±0.05433	1.381±0.06683
48	1.397±0.08548	1.605±0.11	1.795±0.07086	1.705±0.09508
72	1.68±0.09329	1.933±0.1477	2.319±0.1281	2.098±0.08459

MAPRE2 Flow cytometry (figure 3c)

A549+si-con+DDP	A549+si-MAPRE2+DDP	A549/DDP+si-con+DDP	A549/DDP+si-MAPRE2+DDP
21.08±2.021	9.183±1.175	11.09±1.115	11.5±0.9096

Protein stability assay (figure 3f)

	A549/DDP+si-Control	A549/DDP+si-WDHD1
0	98.33±4.778	98.68±4.486
6	9.866±6.049	53.99±7.981
12	10.17±5.697	50.33±8.52
24	0	13.31±4.411

MTT (figure 4a)

	A549/DDP+si-con	A549/DDP+si-WDHD1	A549/DDP+si-WDHD1+DDP
0	1	1	1
12	1.363±0.02589	1.222±0.04801	1.058±0.02613

24	1.577±0.01867	1.472±0.01539	1.174±0.0317
48	1.962±0.01848	1.834±0.0232	1.354±0.03117
72	2.355±0.02632	2.076±0.07811	1.555±0.07287

A549/DDP+si-WDHD1+si-MA+DDP	A549/DDP+si-MA+DDP
1	1
1.185±0.01786	1.156±0.02571
1.408±0.06303	1.341±0.01893
1.674±0.0518	1.538±0.03196
1.895±0.04422	1.757±0.01831

Flow cytometry (figure 4b)

A549/DDP+si-con	A549/DDP+si-WDHD1	A549/DDP+si-WDHD1+DDP
9.203±0.9222	9.003±0.8852	26.6±1.745

A549/DDP+si-WDHD1+si-MAPRE2+DDP	A549/DDP+si-MAPRE2+DDP
9.923±0.837	8.953±1.086

Colony formation assay (figure 4c)

A549/DDP+si-con	A549/DDP+si-WDHD1	A549/DDP+si-WDHD1+DDP
47.82±2.321	50.14±3.55	71.86±2.84

A549/DDP+si-WDHD1+si-MAPRE2+DDP	A549/DDP+si-MAPRE2+DDP
41.45±2.752	38.32±2.35

Wound healing assay (figure 4d)

A549/DDP+si-con	A549/DDP+si-WDHD1	A549/DDP+si-WDHD1+DDP
5.607±0.522	5.633±0.479	2.59±0.5724

A549/DDP+si-WDHD1+si-MAPRE2+DDP	A549/DDP+si-MAPRE2+DDP
5.053±0.5705	5.423±0.347

Animal tumor volume（figure 5a）

	A549/DDP+si-con+DDP	A549/DDP+si-WDHD1+DDP	A549/DDP+si-WDHD1+si-MAPRE2+DDP
0	0	0	0
7	15.48±2.352	2.461±1.375	12.47±0.4627
10	33.1±6.238	6.944±2.865	30.59±1.237
13	67.01±12.41	9.5±2.377	60.94±5.207
16	110.9±10.05	21.75±5.607	99.68±10.1
19	155.7±15.55	52.86±12.74	167.1±11.6
22	263.4±20.26	84.44±13.56	235.1±14.06

25	427.8±25.98	146.8±14.61	400.7±19.97

Immunohistochemistry （figure 6b）

	High	Low
CS(n=10)	10（1/10）	90（9/10）
CR(n=11)	81.8（9/11）	18.2（2/11）

Supplementary

APRC1 PCR(figure Supplementary 1)

A549/DDP	A549
1±0.2537	1.617±0.3973

WDHD1protein sequence

>sp|O75717|WDHD1_HUMAN WD repeat and HMG-box DNA-binding protein 1 OS=Homo sapiens OX=9606 GN=WDHD1 PE=1 SV=1
MPATRKPMRYGHTEGHTEVCFDDSGSFIVTCGSDGDVRIWEDLDDDDPKFINVGEKAYS
CALKSGKLVTAVSNNTIQVHTFPEGVPDGILTRFTTNANHVVFNGDGTKIAAGSSDFLVKI
VDVMDSSQQKTFRGHDAPVLSLSFDPKDIFLASASCDGSVRVWQISDQTCAISWPLLQKC
NDVINAKSICRLAWQPKSGKLLAIPVEKSVKLYRRESWSHQFDLSDNFISQTLNIVTWSP
CGQYLAAGSINGLIIVWNVETKDCMERVKHEKGYAICGLAWHPTCGRISYTDAEGNLGL
LENVCDPSGKTSSSKVSSRVEKDYNDLFDGDDMSNAGDFLNDNAVEIPSFSKGIINDDED
DEDLMMASGRPRQRSHILEDDENSVDISMLKTGSSLLKEEEEDGQEGSIHNLPLVTSQRPF
YDGPMPTPRQKPFQSGSTPLHLTHRFMVWNSIGIIRCYNDEQDNAIDVEFHDTSIHHATH
LSNTLNYTIADLSHEAILLACESTDELASKLHCLHFSSWDSSKEWIIDLPQNEDIEAICL
GQGWAAAATSALLLRLFTIGGVQKEVFSLAGPVVSMAGHGEQLFIVYHRGTGFDGDQCL
GVQLLELGKKKKQILHGDPLPLTRKSYLAWIGFSAEGTPCYVDSEGIVRMLNRGLGNTW
TPICNTREHCKGKSDHYWVVGIHENPQQLRCIPCKGSRFPPTLPRPAVAILSFKLPYCQIAT
EKGQMEEQFWRSVIFHNHLDYLAKNGYEYEESTKNQATKEQQELLMKMLALSCKLERE
FRCVELADLMTQNAVNLAIKYASRSRKLILAQKLSELAVEKAAELTATQVEEEEEEEDFR
KKLNAGYSNTATEWSQPRFRNQVEEDAEDSGEADDEEKPEIHKPGQNSFSKSTNSSDVSA
KSGAVTFSSQGRVNPFKVSASSKEPAMSMNSARSTNILDNMGKSSKKSTALSRTTNNEKS
PIIKPLIPKPKPKQASAASYFQKRNSQTNKTEEVKEENLKNVLSETPAICPPQNTENQRPKT
GFQMWLEENRSNILSDNPDFSDEADIIKEGMIRFRVLSTEERKVWANKAKGETASEGTEA
KKRKRVVDESDETENQEEKAKENLNLSKKQKPLDFSTNQKLSAFAFKQE

MAPRE2 protein sequence

>sp|Q15555|MARE2_HUMAN Microtubule-associated protein RP/EB family member 2 OS=Homo sapiens OX=9606 GN=MAPRE2 PE=1 SV=1
MPGPTQTLSPNGENNNDIIQDNNGTIIPFRKHTVRGERSYSWGMAVNVYSTSITQETMSR

HDIIAWVNDIVSLNYTKVEQLCSGAAYCQFMDMLFPGCISLKKVKFQAKLEHEYIHNFKL
LQASFKRMNVDKVIPVEKLVKGRFQDNLDFIQWFKKFYDANYDGKEYDPVEARQGQDA
IPPPDPGEQIFNLPKKSHHANSPTAGAAKSSPAAKPGSTPSRPSSAKRASSSGSASKSDKDL
ETQVIQLNEQVHSLKLALEGVEKERDFYFGKLREIELLCQEHGQENDDLVQRLMDILYAS
EEHEGHTEEPEAEEQAHEQQPPQQEEY
